# Untapped potential of physiology, behaviour and immune markers to predict range dynamics and marginality

**DOI:** 10.1002/ece3.8331

**Published:** 2021-11-11

**Authors:** Susanne Shultz, Jake A. Britnell, Nicholas Harvey

**Affiliations:** ^1^ School of Earth and Environmental Sciences University of Manchester Manchester UK; ^2^ Chester Zoo Upton‐By‐Chester UK

**Keywords:** conservation, endocrinology, glucocorticoids, gut health, macrophysiology, microbiome, social networks, thyroid hormone

## Abstract

Linking environmental conditions to the modulators of individual fitness is necessary to predict long‐term population dynamics, viability, and resilience. Functional physiological, behavioral, and reproductive markers can provide this mechanistic insight into how individuals perceive physiological, psychological, chemical, and physical environmental challenges through physiological and behavioral responses that are fitness proxies. We propose a *Functional Marginality* framework where relative changes in allostatic load, reproductive health, and behavior can be scaled up to evidence and establish causation of macroecological processes such as local extirpation, colonization, population dynamics, and range dynamics. To fully exploit functional traits, we need to move beyond single biomarker studies to develop an integrative approach that models the interactions between extrinsic challenges, physiological, and behavioral pathways and their modulators. In addition to providing mechanistic markers of range dynamics, this approach can also serve as a valuable conservation tool for evaluating individual‐ and population‐level health, predicting responses to future environmental change and measuring the impact of interventions. We highlight specific studies that have used complementary biomarkers to link extrinsic challenges to population performance. These frameworks of integrated biomarkers have untapped potential to identify causes of decline, predict future changes, and mitigate against future biodiversity loss.

## Graphical abstract

Linking environmental conditions to the modulators of individual fitness is necessary to predict long‐term population viability and resilience. Here, we advocate for a framework that uses multiple integrated biomarkers to establish functional changes. This framework has untapped potential to identify causes of decline, predict future changes, and mitigate against future biodiversity loss.

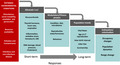

## 1 INTRODUCTION

One in five vertebrate species is classified by the IUCN as vulnerable, endangered, or critically endangered (Hoffmann et al., 2010). These declines and losses are largely attributed to anthropocentric changes in the environment such as land conversion, climate change, and unsustainable natural resource harvesting and extraction (Brook et al., 2008). Ultimately, range contraction and biodiversity loss are the end product of extrinsic or intrinsic challenges leading to population decline, emigration, and local extirpation. Across a species’ range, populations occur along ecological gradients from optimal, or central, habitats, where conditions and resources lead to high population density or maximal reproduction and survival, to marginal habitats where population density, reproduction, and/or survival are much lower (Holt, 2009; Kawecki, 2008). Identifying and mitigating the causes of reduced reproduction, compromised survivorship, and emigration are key for predicting and arresting biodiversity loss (Chown & Gaston, 2008).

The simplest species models assume that fitness follows a unimodal distribution with high density and growth rates in the center of a range and low density or poorly performing marginal populations found at the range periphery (Guo et al., 2005). However, environmental characteristics and species’ responses are much patchier than this, such that geographic and ecological marginality are not equivalent (Pironon et al., 2017; Santini et al., 2019). Variation in carrying capacity across environmental gradients can lead to high‐density “source” populations producing emigrants that disperse to low‐density “sinks” in marginal habitats (Pulliam & Danielson, 1991); however, if all populations reach carrying capacity, dispersal is likely to be balanced between high‐ and low‐density populations (Fretwell, 1969, 1972). In natural populations, however, environmental and demographic stochasticity result in dynamic reproduction, survival, and dispersal rates (Holt, 2003), which can cause low‐density populations to be less resilient, with higher rates of local extirpation and recolonization, than high‐density populations. Moreover, environmental change can either increase carrying capacity, leading to population growth and colonization, or result in decreased carrying capacity, population decline, local extirpation, and range contraction (Gaillard et al., 2000). Where ecological conditions are extreme for a species, local extirpation occurs faster than recolonization, limiting viable ranges. Range contraction occurs where previously resilient populations become unviable as growth and immigration rates no longer sustain the population. Identifying and predicting these dynamics in marginal populations provides key insight into long‐term dynamics.

Responses to environmental change can be predicted using climate envelope, population viability, and mechanistic distribution models. Climate envelope, or habitat suitability, models relate species occurrence to environmental variables to explain or predict species distribution (Pearson & Dawson, 2003) and can predict occupancy changes under different environmental scenarios. While they are widely applicable because they require limited information, climate envelope models have limited capacity to predict local occupancy change as they do not incorporate population specific dynamics, carrying capacities, species interactions, or dispersal potential. Climate envelope models based solely on occupancy are often poor predictors of habitat suitability and patterns of population abundance across ranges (Osorio‐Olvera et al., 2019). Population viability models, by contrast, can reliably predict future trends for specific populations (Brook et al., 2000) but require accurate vital rates, which are labor‐ and time‐intensive to collect. Thus, their ability to predict resilience and viability across taxa and at large scales is limited. A middle ground is combining range and population dynamics for large‐scale assessments of occupancy based on factors associated with local resilience or vulnerability. Mechanistic population and distribution models can provide this link between local population viability and range dynamics (Kearney & Porter, 2009) by using key behavioral and ecophysiological factors as functional indicators of resilience. Such models are more widely applicable than population viability analyses, are more robust, can be extrapolated to other populations, and have more predictive value than climate envelope models. They can also provide rapid and large‐scale population assessments of marginal habitats to produce spatially explicit, predictive distribution maps across ecological gradients.

Interpreting the relevance of functional indicators across ecological gradients requires understanding their relationship with population responses (Bonier et al., 2009). Here, we advocate a *Functional Marginality* framework using physiological and behavioral indicators to assess population resilience. First, we describe functional physiological and behavioral indicators in the context of key stressors and explain methods to incorporate multiple indicators in predictive models. Second, we describe how functional marginality can be used to identify predictive hypotheses for occupancy changes, range dynamics, responses to environmental change, and evaluate the efficacy of management interventions. Although we primarily focus on mammals, this approach could be applied to many other taxa.

### 1.1 Functional indicators

Functional traits are morphological, physiological, or behavioral traits that are fitness proxies via their effects on growth, reproduction, and survival (Violle et al., 2007), and indicate how a species perceives and responds to its environment (McGill et al., 2006). Positive functional indicators include relaxed time budgets, positive energy balance, and surplus energy stores manifesting in good body condition, good reproductive performance, and sound immune function or low disease burden. Negative indicators are those associated with a decline in condition in response to four types of challenges or stressors: physiological, psychological, chemical, and physical (Pottinger, 2003). Physiological stressors include resource, nutrient, or water restriction and disease. Psychological stressors include conflict, predation risk, and disturbance or persecution. Chemical stressors include altered pH, low dissolved oxygen, and exposure to pollutants, contaminants, or toxins. Finally, physical stressors encompass climate extremes and substrate as well as damage incurred by predation, conflict, or injury. Each class of stressor is associated with characteristic physiological and behavioral responses tied to pathways that maintain homeostasis (Madliger et al., 2018). Here, we discuss how physiological, psychological, chemical, and physical stressors can be manifest in physiological and behavioral indicators.

#### 1.1.1 Physiological stressors

##### Energetic and metabolic stress

Fitness is inextricably tied to maintaining sufficient energy reserves to support metabolism, invest in reproduction, and allow individuals a buffer during periods of scarcity or in response to challenges or disease (Burger et al., 2019). Responses to stressors often incur an energetic cost, which can compromise reproduction or growth (Christiansen et al., 2013). In response to a decline in resource availability an organism can change its behavior to increase energy availability by increasing foraging rate, feeding time, or travel distances, before mobilizing energy reserves or down regulating metabolism (Reneerkens et al., 2002). Thus, environmental change due either to climate or land use can have direct impacts on resource availability and seasonality. Behavioral changes can indicate energy budget challenges. For example, animals can adjust time budgets to spend more time traveling and feeding, and less time resting, to meet energy needs (Dunbar et al., 2009). Changes in habitat use or diet can also indicate energetic stress. For example, browsers becoming more dependent on grazing (Landman et al., 2013), or conversely grazers becoming more reliant on browse (Faith, 2012) suggesting a forced shift from preferred foods. The extent of temporary seasonal switching versus prolonged dependence on less preferred “fallback foods” can indicate significant resource stress in marginal habitats (Grueter et al., 2009). This is especially true when animals are pushed from an optimal diet to consume items that they are not physiologically adapted to handle (Ingala et al., 2019; Kitaysky et al., 2006). Thus, changes in the dynamics of seasonal and prolonged dietary shifts within and between populations could be used as a proxy for energetic stress. For terrestrial vertebrates, in addition to food limitations, water stress caused by abstraction or seasonality can lead to changes in space use, increased aggregations, and distance traveled.

Energy stress is also manifest in physiological responses. The hypothalamus–pituitary–thyroid axis (HPT) regulates metabolic rate by changing the amount of circulating thyroid hormone in response to metabolic requirements and responds to both thermal stress and food availability (Costa‐e‐Sousa & Hollenberg, 2012). Thyroid hormones and metabolic rate measures can identify how quickly animals are mobilizing and using energy; however, opposing responses to thermal and nutritional challenges can lead to a difficult to interpret metabolic trade‐off between energy use and acquisition (Cristóbal‐Azkarate et al., 2016). Large, longer‐term differences in energy balance can be evaluated through changes in body condition, as the loss of muscle and fat reserves suggests a negative energy budget. Body condition scoring is routinely used in the management of wild mammals and standardized schemes have been developed for several species including black rhinos (*Diceros bicornis*) (Reuter & Adcock, 1998) and African buffalo (*Syncerus caffer*) (Ezenwa et al., 2009). Despite thyroid hormones offering a window into an individual's energy balance (Behringer et al., 2018), fewer studies utilize thyroid hormones as biomarkers to assess the impact of environmental factors on fitness than those that use glucocorticoids, which indicate acute fluctuations in energy mobilization.

The impact of resource driven dietary shifts and external stressors can also be manifest within the gut, where microbial communities perform key functional roles in the host and contribute significantly to host health (Gilbert et al., 2018; Sommer & Bäckhed, 2013). Diet changes can lead to changes of key microbiota, which impact on gut function (Borbón‐García et al., 2017). Beyond diet, microbiome communities are influenced by a range of factors including habitat, social network properties, and climatic conditions (Trevelline et al., 2019). Furthermore, primary and secondary acute stress responses such as glucocorticoids modulate the microbiome (Noguera et al., 2018). An imbalance of the microbial community, known as dysbiosis, can reduce digestive efficiency, increase inflammation, and susceptibility to infection (Amato et al., 2013; Dethlefsen et al., 2007; Gilbert et al., 2016). Signatures of dysbiosis will vary across hosts, as microbiome composition is sensitive to both diet and vertical transmission; however, dysbiosis or atypical microbiomes can be characterized by the degree of a displacement from a core microbiome composition (Zaneveld et al., 2017). Although the fitness consequences of changes in microbial community are poorly understood, microbiome composition has been linked to reproductive performance (Antwis et al., 2019) and cellular inflammation (Walshe et al., 2019).

##### Acute challenges: predation, disturbance, and social instability

A primary response to acute stressors such as predation, persecution, or disturbance is the activation of the hypothalamic–pituitary–adrenal (HPA) axis in birds and mammals or the hypothalamic–pituitary–interrenal (HPI) axis in fish, amphibians, and reptiles, which leads to the release of glucocorticoids (GCs) and catecholamines (Beehner & Bergman, 2017; Sopinka et al., 2016). Thus, the HPA/HPI axes and epinephrine stress responses are coupled with metabolism and metabolic rates, as both increase the body's ability to mobilize energy for acute challenges. GCs have been used as an indicator of stress, commonly under the assumption that chronic elevation compromises health and ultimately fitness (Millspaugh & Washburn, 2004). However, short‐term activation of the HPA is an adaptive response to allow individuals to effectively respond to acute challenges such that relationships between GCs and fitness are not straightforward (Moberg, 2000). The relationship between GC levels, GC reaction potential, and individual fitness (Bonier et al., 2009; Breuner et al., 2008) is context‐dependent, such that during good conditions a high GC responsiveness is associated with poor survivorship and recruitment, whereas during poor conditions the relationship may be reversed when individuals in poor condition become unable to mount significant GC responses (Blas et al., 2007). There is so much variation in how individuals and species respond to chronic stress that there is no consistent profile to identify chronic stress across species (Dickens & Romero, 2013). What GCs do provide is evidence for a perceived challenge or stressor.

Combining physiological responses with other functional traits can help identify where these responses may lead to reduced fitness. For example, behavioral and endocrine profiles can be supplemented with direct physiological measures such as blood pressure, heart, and respiratory rate, if these are feasible for the study species, or proxies for these metrics if they are not (Madliger et al., 2018; Sopinka et al., 2016). Social instability also interacts with physiology (Gersick & Rubenstein, 2017; Seebacher & Krause, 2017) and is associated with elevated GCs in spotted hyenas (*Crocuta crocuta*) (Van Meter et al., 2009), Barbary macaques (*Macaca Sylvanus*) (Edwards et al., 2013), olive baboons (*Papio anubis*) (Sapolsky, 1992), and horses (Nuñez et al., 2014). Human disturbance can also disrupt the normal behavior of animals such as flight responses or changes in space use and time budgets (Wong & Candolin, 2015). The key to understanding the impact of all these markers is how they impact on fitness proxies such as energy reserves, reproductive, and survival rates.

##### Disease burdens

Heavy disease or parasite burden have fitness consequences (Pedersen & Fenton, 2007) including survival and fecundity that directly impact on population dynamics (Hillegass et al., 2010; Hudson, 1986; Hudson et al., 1998). Gastrointestinal nematode communities, or the nemabiome, can directly affect host fitness but also have the potential to influence resistance and susceptibility to other infecting species (Supali et al., 2010). However, parasite infections are not universally harmful, removing helminths induces a strong inflammatory response (Walshe et al., 2019) and can potentially trigger autoimmune diseases (McKay, 2009). In addition to direct transmission risk, widespread anthropogenic disturbance can exacerbate disease risk through stress‐induced immunosuppression. Although there is limited causal evidence between human impacts, stress, and disease occurrence, it is widely assumed that stress may be a major cause of increased susceptibility to wildlife disease (Hing et al., 2016). This may be due to the suppression of reproduction and immune function by the HPA axis as evidence for direct relationships between elevated GCs and parasite burden is well established (O'Dwyer et al., 2020).

Immune responses are also molecular indicators of physiological challenge or stress (Celi et al., 2019; Madliger et al., 2018; Sopinka et al., 2016). Immunoglobulins, or “antibodies” (e.g., IgA, IgG, IgM), form a critical part of the immune response by recognizing, binding to and neutralizing antigens, such as bacteria or viruses (Schroeder & Cavacini, 2010). Fecal antibody assays have been used to measure the immune response to parasites (Watt et al., 2016), which in turn correlate with survival (Sparks et al., 2018). Additional biomarkers that are associated with short‐term and long‐term responses to external challenges and stressors are blood parameters such as hematocrit levels and white blood cell counts (Madliger et al., 2018; Sopinka et al., 2016). Reduced hematocrit levels in birds are associated with a range of challenges including disease burden and nutritional status (Fair et al., 2007). Heterophil or neutrophil to lymphocyte ratios can indicate chronic stress, whereas eosinophil levels can indicate infectious disease (Davis et al., 2008). Inflammation markers can provide evidence of infectious and noninfectious processes. Calprotectin, lipocalin, and lactoferrin are inflammation markers that limit bacterial growth (Mao et al., 2012) and are used to diagnose inflammatory bowel disease in humans (Van Rheenen et al., 2010). Such biomarkers, which are gaining traction in human clinical practice, have untapped potential for use in wildlife monitoring. Increased metabolism results in the production of chemically reactive metabolic by‐products known as reactive oxygen species (ROS) (Sies, 1991). Typically, ROS are removed from the body by antioxidants, but if they are generated in excess, oxygen radicals build up and bind to a range of biological molecules. This oxidative stress results in cellular and DNA damage, reduced defense mechanism, and accelerated aging (Finkel & Holbrook, 2000). Chronically elevated GC production is associated with oxidative stress across species (Costantini et al., 2011).

#### 1.1.2 Chemical and physical stressors

In addition to natural stressors, organic compounds, trace elements, and pharmaceuticals have all been responsible for catastrophic species declines (Rowe, 2008). Chemicals that are persistent and can bioaccumulate in food webs are particularly dangerous as they can have destabilizing effects on ecosystems. Major environmental contaminants are pesticides, perfluorinated compounds, and pharmaceuticals. Bioaccumulation of persistent organochlorines, such as DDT and associated compounds, has been implicated as major environmental contaminants, which cause catastrophic bird declines and are implicated in endocrine disruption in humans (Blus, 2011). Tributyltin (TBT) is an antifoulant that is well known for its endocrine disruptive effects. Although DDT and TBT are now banned globally, their persistence means that they still occur at appreciable levels in the environment. Perfluoroalkylated compounds are commonly used in various forms of manufacturing. They are persistent in the environment and are linked to endocrine disruption, fertility, and metabolism (Jensen & Leffers, 2008). These compounds also affect human health, for example, increasing cancer risk, and declines in reproductive health, and longevity. The widespread use of antibiotics for human and veterinary health is linked to environmental bioaccumulation that, in turn, is implicated in the spread of antimicrobial resistance (Singer et al., 2016). Anti‐inflammatory drugs also bioaccumulate with detrimental effects. The unregulated veterinary use of the anti‐inflammatory drug diclofenac resulted in catastrophic declines of Asian vulture populations (Green et al., 2004; Shultz et al., 2004). Marine predators are especially vulnerable due to biomagnification and coastal habitats are particularly vulnerable to bioaccumulation due to sewage, run‐off, and sedimentation. For this reason, seabirds have been touted as sentinels for estuarine and continental shelf habitats (Burger & Gochfeld, 2004). Thus, the potential role and impact of chemical contaminants on fitness should be evaluated in unexplained population collapse and range contraction, especially where changes in resource availability, disease, or acute stressors do not appear sufficient to explain declines.

Physical stressors such as injury, particularly when associated with pain, are associated with increased glucocorticoid levels in free ranging mammals (Ganswindt et al., 2010; Rolland et al., 2017; Tripp et al., 2011; Wolf et al., 2018) and birds (Scheun et al., 2021). In some species, physical injury is a major cause of morbidity and mortality. For example, marine mammals including whales, seals, and manatees are particularly vulnerable to anthropogenic injury. In fact, >95% of Florida manatee adults show evidence of boat strike injury (Bassett et al., 2020). Seabirds are also subject to high rates of anthropogenic injury (Dias et al., 2019). Critically, in addition to direct mortality, injury is associated with reproductive suppression and delayed mortality in birds (Fajardo et al., 2000; Parsons et al., 2018), fish (Mueller et al., 2020), and reptiles (Sack et al., 2017) and can lead to population decline associated with high levels of physical injury. Thus, although physical injury is not necessarily a widespread problem, in some species both the acute and chronic impacts of injury are significant conservation challenges.

### 1.2 Interpreting and integrating indicators

Using functional markers at large scales to identify vulnerable or declining populations requires reference or benchmark values from well performing populations. Where this is not possible, for example, in a species undergoing widespread declines and range collapse, it may be possible to use benchmarks from historical records or use congeners as a reference population (Britnell et al., 2021; Bocherens et al., 2015; Kerley et al., 2012). The expectation is that individuals from populations in marginal habitats or under challenges will have either single or multiple functional indicators that diverge from an optimal benchmark. Negative indicators will increase and positive indicators will decrease with the distance from central or optimal habitats (either geographically or in terms of niche hypervolume).

Single marker studies can give an incomplete or even misleading picture of individual condition and population health as multiple stressors can act independently or in tandem causing additive, synergistic, or antagonistic effects (Beldomenico & Begon, 2010; Todgham & Stillman, 2013). Few studies employ multitool approaches to evaluate the impact of stress on multiple physiological pathways (Madliger et al., 2018) and studies, which investigate stressors, physiology, and demography together are even more scarce (Beehner & Bergman, 2017). Functional responses to multiple environmental challenges can be measured using the concept of allostatic load, which is the cumulative physiological impact of challenges, when the body can no longer buffer challenges this becomes allostatic overload (McEwen & Wingfield, 2003). Allostatic overload leads to loss of condition, immune, and reproductive suppression and disease.

**FIGURE 1:**
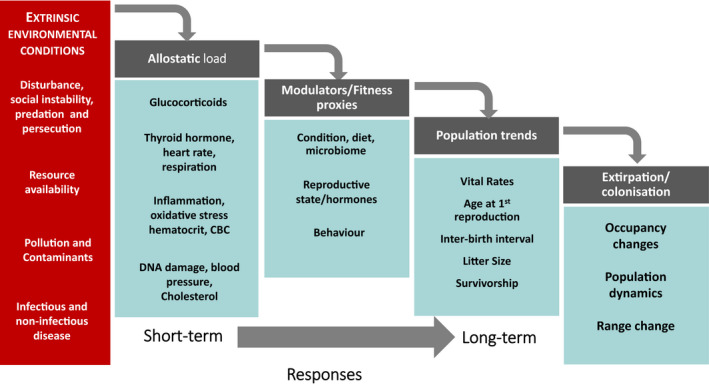
A conceptual diagram showing the different biomarkers available that can be integrated into studies using the footprints and pathway approach

**FIGURE 2:**
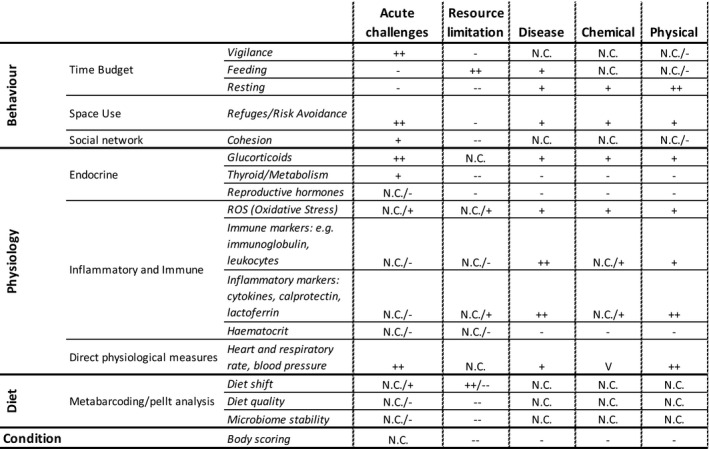
Conceptual framework for testing alternative hypotheses for different stressors. +/− indicates potential direction of change. ++/−− indicators are expected to show large magnitude responses. N.C. indicates no consistent/predictable response

Concurrently evaluating a suite of biomarkers can provide information about how the different pathways interconnect and impact fitness in relation to environmental stressors or challenges (Figure 1, Table 1). The relationship between multiple biomarkers and population performance can be evaluated with a multivariate model selection approach (Johnson & Omland, 2004), a growth curve model or similar structural equation modeling approaches (Schlotz et al., 2011), or multidimensional data analysis. Thus, a set of indicators can be used to set up alternative hypotheses to determine primary challenges causing poor performance (Figure 2). For example, acute stressors such as disturbance, predation, or persecution may be associated with space use or time budget changes (use of refuges or cover, increased vigilance, movement, and decreased feeding and/or resting) and increased HPA activation. Resource limitation should be associated with increased foraging effort, diet changes, and decreased metabolic rate. Diet changes can be manifest by either increased switching to low‐quality “fall back” foods during times of scarcity, or in extreme cases, the diet being completely comprised of low‐quality items. Loss of condition and fitness costs, such as decreases in survival and fecundity, that are not associated with diet or behavioral change will likely be caused by disease when there are clear inflammatory responses, and by contaminants or chemical stressors when there are not. As each vulnerable population may face a unique set of challenges, a predefined set of testable hypotheses can be used to identify most likely candidates. A contingency table of expected responses can act as a starting point for formulating testable hypotheses based on the Functional Marginality Framework (Figure 2).

**TABLE 1:** Example studies linking environmental stressors with physiological responses and demographic/population consequences

Class	Species	Challenge	Functional indicators	Population/fitness proxies	Reference
Physiological‐ Resource	Killer whales *Orcinus orca*	Fish abundance Vessel density	fGCs, fT3	Pregnancy ↓ Population growth ↓	Wasser et al. (2017)
	African elephants *Loxodonta africana*	Rainfall	fGCs fPG	Reproductive function ↓	Foley et al. (2001)
	Shetland ponies *Equus caballus*	Seasonality	Heart rate Locomotion T3 ↑	Field metabolic rate ↑	Brinkmann et al. (2016)
	Soay sheep *Ovis aries*	Maternal effects Genetic variation	Ig proteins Fecal egg count	Survival ↓	Sparks et al. (2018)
	Roe deer *Capreolus capreolus*	Primary productivity	fA, fPG, Estradiol fGCs, fN, IgA	Reproductive condition ↓	Escribano‐Avila et al. (2013)
	Cape Mountain zebra *Equus zebra zebra*	Season	GCs ↑ Diet shifts	population growth rate ↓	Lea et al. (2018)
	Barbary macaques	Seasonality‐food availability	T3 ↑		Cristóbal‐Azkarate et al. (2016)
	White‐tailed deer *Odocoileus virginianus*	Seasonality‐food availability	T3/T4		Bahnak et al. (1981)
	European badger *Taxidea taxus*	Food availability	T3		Harlow and Seal (1981)
	Chimpanzee *Pan troglodytes*	Habitat quality	Creatinine, GCs		Wessling et al. (2018)
	Common vampire bats *Desmodus rotundus*	Habitat conversion	Diet, behavior, microbiome	Immune function	Ingala et al. (2019)
	Black howler monkey *Alouatta pigra*	Logging and deforestation	Diet and microbiome diversity		Amato et al. (2013)
	Primates	Habitat quality	Microbiome diversity		Stumpf et al. (2016)
	Western fence lizard	Central‐peripheral populations	GCs, plasma protein, hematocrit	Body weight	Dunlap (1995), Dunlap and Wingfield (1995)
	Maned wolf *Chrysocyon brachyurus*	Transformed landscapes	GCs ↑, T3↑, PG↓	Suggested reduced reproduction	Vynne et al. (2014)
	Primates (*Pan* spp, *Ateles* spp, *Papio*)	Habitat quality	Time budgets	Range and occupancy dynamics	Bettridge et al. (2010), Dunbar et al. (2009), Korstjens and Dunbar (2007), Korstjens et al. (2010)
Physiological‐ Acute Stress	Guadalupe fur seals	Capture	Aldosterone ↑ return to baselines		DeRango et al. (2019)
	Bottle‐nosed dolphin	Beaching	Aldosterone ↑		Champagne et al. (2018)
	Stingrays	Tourist activity	ROS ↑		Semeniuk et al. (2009)
	Damselfly *Enallagma cyathigerum*	Predation	Stress proteins O^2^ consumption Enzyme activity Oxidative stress	Growth rates ↓	Slos and Stoks (2008)
	Black grouse *Tetrao tetrix*	Human disturbance	Feeding times ↑	Energy expenditure ↑	Arlettaz (2015) #1790
	Great tit *Parus major*	Artificial light	Corticosterone ↑	Fledging ↓	Ouyang et al. (2015)
	Eastern black rhinos	Captive environment	PG↓ androgens↓	Reproduction↓	Antwis et al. (2019), Edwards et al. (2015)
	Florida manatee *Trichechus manatus latirostris*	Release from rehabilitation; injury and disease	Serum and urinary creatinine ↑, creatine kinase ↑, urea nitrogen, GCs↑, lymphocyte proliferation ↓		Manire et al. (2003), Tripp et al. (2011)
	African elephants *Loxodonta africana*	Translocation	GCs ↑		Jachowski et al. (2013), Viijoen et al. (2008)
	Chimpanzee Pan troglodytes	Human Disturbance	GCs ↑		McLennan et al. (2019)
Disease	Red grouse	Nematodes		Fecundity ↓	Hudson (1986), Hudson et al. (1998)
	Seychelles warblers (*Acrocephalus sechellensis*)	Parasitism, habitat quality	ROS ↑	Survival and fecundity↓	van de Crommenacker et al. (2011), van de Crommenacker et al. (2012), van de Crommenacker et al. (2017)
	Soay sheep	Parasitism	IgA	Survival ↓	Sparks et al. (2018), Watt et al. (2016)
Chemical and Physical	Fathead minnow *Pimephales promelas*	Environmental estrogen EE2		Survival and fecundity↓	Schwindt et al. (2014)
	Little auk	Mercury exposure		Body condition↓growth rate ↓	Amélineau et al. (2019)
	Black legged kittiwakes	Perfluorinated carboxylates	GC ↓	Body condition↓ Hatching↓	Tartu et al. (2014)
	Monk seals	lethal injury	GC, T3	Body condition↓	Gobush et al. (2014)
	African elephants *Loxodonta africana*	Foot injury	fGC↑	Body condition↓	Ganswindt et al. (2010)

Note

We highlight studies that link environmental challenges with multiple biomarkers and fitness proxies in terms of health, condition, or reproduction.

Multivariate modeling approaches can tease apart the relative importance of extrinsic factors. The role of environmental traits and functional indicators of health outcomes (i.e., reproductive failure, elevated mortality) can be evaluated using a model selection approach (Deelen et al., 2019). Multiple markers can also effectively evaluate the extent of “dysfunction” as a measure of deviation, such as Mahalanobis distance, from a multivariate central tendency (Milot et al., 2014). Evaluating the model weight for different factors (Johnson & Omland, 2004) can identify functional indicators that best predict fitness variance or population resilience, which can be used as key population health markers and focused on in future research. Clearly, a challenge with this macrophysiological approach is identifying markers that can be rapidly and noninvasively collected such as demography, behavior (association patterns, space use, and time budgets), and noninvasive biological samples (e.g., fecal and urine). Using model species, where noninvasive samples can be easily collected from a large number of individuals and tied to reproductive, survivorship, or population growth rate outcomes (Lea et al., 2018), is a key priority for developing a macrophysiological approach.

### 1.3 Theoretical frameworks

**FIGURE 3:**
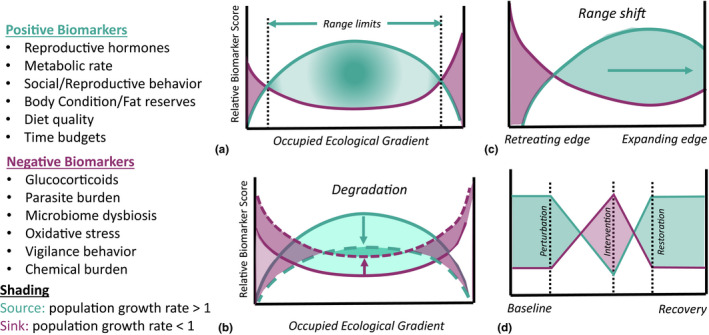
Conceptual diagram of the *Functional Marginality* Framework. (a) Viable populations are determined by good functional condition leading to sustainable growth rates, range limits are determined by an increased burden of negative functional traits relative to positive ones. (b) Range shifts will be associated with improving functional condition on the expanding edge and declining condition on the retreating edge. (c) Habitat degradation leads to a net decline in functional condition (balance of positive indicators and negative allostatic load) across occupied habitat resulting in more sink populations and fewer source populations. (d) Functional condition can be tracked over time by repeatedly measuring positive and negative functional traits, and will exhibit characteristic profiles during periods of threat and recovery

Mechanistic distribution models use functional traits to link environmental variation with individual‐ and population‐level performance (Buckley et al., 2010) as variation in physiological biomarkers of stress, health, and reproduction can act as these heuristic indicators of population viability (Chown & Gaston, 2008; Ellis et al., 2012; Gaston et al., 2009). Thus, they can predict likely population performance and range dynamics including the probability of colonization and extirpation under changing conditions (Figure 3a). For example, range‐wide land transformation and climate changes can lead to an increased allostatic load (e.g., oxidative stress, inflammation, and disease) and a decline in positive biomarkers (reproductive function, metabolism, hematocrit levels) in adversely affected populations, with a net reduction in functional condition (Figure 3b). Functional traits can also evaluate patterns of range contraction, where the expanding edge will be associated with improved functional traits and the retreating edge associated with declines in positive indicators and increasing burden of negative indicators (Figure 3c). Moreover, if range contraction is characterized by retreat into suboptimal refuges away from encroaching threats (Scheele et al., 2017), then in additional to poor functional condition in the contracting edge, remaining populations should have poor functional condition as compared to populations in optimal habitats, or where baselines are not available, relative to closely related species (Figure 3c). If environmental change causes species to retreat into optimal habitats (Channell & Lomolino, 2000), then functional traits in the remaining habitats should be consistent with those in optimal habitats or source populations. We would also expect a truncated distribution of markers toward less optimal states across the remaining range (Figure 3c).

Although variation in resource availability across landscapes is widely appreciated, metabolic costs also vary in terms of slopes, substrates, and thermal stresses (Shepard et al., 2013). Incorporating spatial patterns of energy availability and costs in “energetic landscapes” can provide a step change in our understanding of how environmental conditions impact on fitness at the individual and population level. Spatial variation in threats from predation, disturbance, and disease risk can be used to create predictive models of “landscapes of fear” and “landscapes of disgust” (Gallagher et al., 2017; Laundré et al., 2001; Weinstein et al., 2018). In particular, spatial variation in predation risk has consequences on physiology, reproduction, immune function, and behavior (Clinchy et al., 2011, 2013). These spatial models can be integrated to create “landscapes of stress,” where physiological or behavioral trade‐offs can be directly incorporated into population or habitat use models (Koprivnikar & Penalva, 2015). For example, brown bears (*Ursus arctos)* near human settlements have lower heart rate variability, a cardiovascular indicator of stress, and they move further during increased human activity, which is expected to have an energetic cost (Støen et al., 2015). Similarly, landscape features, movement, and disturbance together predict physiological state in grizzly bears (Wilson et al., 2021).

We can also use indicators to test intervention success or the functional recovery of individuals or a population, which can provide insights into efficacy of restoration, colonization, and reintroduction. As humans have extensively changed and degraded habitats, conservation efforts often try to restore habitats or populations to reflect a historical state or ecological baseline (Britnell et al., 2021). A mechanistic approach can provide the evidence about how best to restore or manipulate degraded systems and how to establish whether an intervention has had the desired response (Hobbs et al., 2014). Successful interventions should increase population growth rates and nudge a population from being a sink to being self‐sustaining or a source. Following an intervention, negative biomarkers should decrease, and positive biomarkers increase, relative to pre‐intervention levels (Figure 3d; Cooke & Suski, 2008). The relationship between functional indicators and fitness can be assessed through changes in vital rates, for example, by monitoring changes in pregnancy rates of vertebrates before and after restoration or policy implementation (Pallin et al., 2018).

Planning for, and the short‐ and long‐term effects of, active management such as handling, translocation, and reintroduction can also be evaluated with functional indicators. Factors such as climate suitability are predictors of translocation failure (Bellis et al., 2020), and mechanistic distribution models can increase the robustness of habitat suitability predictions. Translocation success is also linked to stress responses and resilience, which occur during the translocation event and the establishment phase immediately after release (Dickens et al., 2010). Following an intervention, positive and negative biomarkers should return to pretranslocation levels after intervention and recovery. Conducting physiological monitoring before, during, and after release can improve our understanding of translocations, and the factors required for them to be a success. For example, a study using the Florida manatee *(Trichechus manatus latirostris)* in a simulated release process found that decreased food intake and changes in water salinity led to serum creatinine elevations and impaired immune function, indicated by lymphocyte proliferation assays (Manire et al., 2003). This is beneficial from a conservation perspective, as it increases the probability of future reintroduction success, and from a welfare perspective, as it allows methods to be refined to avoid stress and reduces the number of animals required (Tarszisz et al., 2014).

### 1.4 Examples

There is now a small but growing number of studies that have used functional traits to understand range dynamics and differences between central and peripheral populations. For examples, GCs, blood parameters, and body condition vary between central and marginal populations of Western fence lizards (*Sceloporus occidentalis*) (Dunlap, 1995; Dunlap & Wingfield, 1995). Combining variation in time budgets with species distribution models in primates is an excellent example of using behavior patterns to understand drivers of population and range dynamics (Bettridge et al., 2010; Dunbar et al., 2009; Korstjens & Dunbar, 2007; Korstjens et al., 2010). There is also extensive evidence for how pollution and contaminants affect fitness proxies and functional indicators in birds (Rattner et al., 1984), although this approach has not been widely used to evaluate spatial range dynamics in a macroecology context. Despite this, there are limited examples of studies that evidence links between environmental stressors, physiology, behavior, and fitness measures to predict both individual‐ and population‐level responses to challenges (Beehner & Bergman, 2017; Cooke et al., 2013). We have summarized a range of studies that have used functional markers to assess the impact of challenges and population or fitness consequences (Table 1). There are, however, a few key studies that have evaluated links between environmental challenges, functional markers, and population‐level variation in resilience and viability. Physiological and behavioral biomarkers have been used to identify marginal or “refugee” populations in Cape mountain zebra (*Equus zebra zebra*) (Lea et al., 2018) where poor reproduction performance is associated with elevated androgens and glucocorticoids as a consequence of resource limitation and skewed population sex ratios. Functional markers including elevated creatinine, C‐peptide, and glucocorticoids were used to evidence thermal stress in chimpanzees (*Pan troglodytes*) inhabiting a savannah‐mosaic habitat at the margins of their range (Wessling et al., 2018). Hawaiian monk seals (*Neomonachus schauinslandi*) have experienced declines since the 1950s that have been variously attributed to poor juvenile survival due to resource limitations, injury, and disease (Harting et al., 2021). Declining subpopulations were associated with chronic elevation of fGCMs and low fT3, especially in immature individuals and had, on average, poorer survival rates and lower intrinsic population growth rates (Gobush et al., 2014). In better performing populations, multiple markers still highlighted how anthropogenic injury and disease relative to malnutrition affected intrinsic growth rates (Harting et al., 2021). This modeling approach that identifies how local stressors affect growth rates could be applied to most conservation scenarios.

## 2 CONCLUSION

In recent decades, much research has been carried out to develop biomarkers, which provide an indication of how the environment affects the physiological and behavioral state of an organism and ultimately on fitness. This is a difficult task as physiology is extremely complex. Physiological responses are the result of multiple interconnecting pathways, which can respond to the same stressors and interact with each other, making the change in a single biomarker difficult to relate to fitness. We propose that the establishment of complementary and integrated biomarkers to indicate population health, properly validated and applied to testable hypotheses, would be a major advance for large‐scale ecology and conservation. Validation, the discovery of relevant biomarkers or combination thereof, is a key part of this approach. The approaches we describe can be used to show which biomarkers are useful at predicting future changes in fitness measures associated with population changes. Once established, these biomarkers can be the basis for investigating the causes of poor individual health and changes in survival and reproduction and testing ecological and conservation hypotheses. This information can help to uncover the causes of distributional limits and predict future changes, estimate resilience of populations to novel threats, assess the efficacy of conservation efforts, and reveal macroecological trends and processes. This approach provides conservation biologists and practitioners the ability to produce evidence for the causal mechanisms underlying conservation problems and macro‐ or evolutionary ecologists the ability to investigate the physiological mechanisms underlying long‐term and large‐scale processes. Advances in these fields can contribute toward the calls for evidence‐based conservation and help to alleviate the threat of species extinctions and ecological collapse.

## CONFLICT OF INTEREST

We declare there are no conflicts of interest associated with this article.

## AUTHOR CONTRIBUTIONS

**Susanne Shultz:** Conceptualization (equal); Project administration (lead); Supervision (lead); Writing‐original draft (equal); Writing‐review & editing (lead). **Jake A. Britnell:** Conceptualization (equal); Writing‐original draft (equal); Writing‐review & editing (supporting). **Nicholas Harvey:** Writing‐original draft (supporting); Writing‐review & editing (supporting).

## Data Availability

There are no primary data presented in this manuscript. All studies discussed are referenced in the manuscript.

## References

[ece38331-bib-0001] Amato, K. R. , Yeoman, C. J. , Kent, A. , Righini, N. , Carbonero, F. , Estrada, A. , Rex Gaskins, H. , Stumpf, R. M. , Yildirim, S. , Torralba, M. , Gillis, M. , Wilson, B. A. , Nelson, K. E. , White, B. A. , & Leigh, S. R. (2013). Habitat degradation impacts black howler monkey (*Alouatta pigra*) gastrointestinal microbiomes. The ISME Journal, 7, 1344–1353. 10.1038/ismej.2013.16 23486247 PMC3695285

[ece38331-bib-0002] Amélineau, F. , Grémillet, D. , Harding, A. M. , Walkusz, W. , Choquet, R. , & Fort, J. (2019). Arctic climate change and pollution impact little auk foraging and fitness across a decade. Scientific Reports, 9, 1–15. 10.1038/s41598-018-38042-z 30705325 PMC6355795

[ece38331-bib-0003] Antwis, R. E. , Edwards, K. L. , Unwin, B. , Walker, S. L. , & Shultz, S. (2019). Rare gut microbiota associated with breeding success, hormone metabolites and ovarian cycle phase in the critically endangered eastern black rhino. Microbiome, 7, 27. 30770764 10.1186/s40168-019-0639-0 PMC6377766

[ece38331-bib-0004] Bahnak, B. R. , Holland, J. C. , Verme, L. J. , & Ozoga, J. J. (1981). Seasonal and nutritional influences on growth hormone and thyroid activity in white‐tailed deer. The Journal of Wildlife Management, 45, 140–147. 10.2307/3807882

[ece38331-bib-0005] Bassett, B. L. , Hostetler, J. A. , Leone, E. , Shea, C. P. , Barbeau, B. D. , Lonati, G. L. , Panike, A. L. , Honaker, A. , & Ward‐Geiger, L. I. (2020). Quantifying sublethal Florida manatee watercraft interactions by examining scars on manatee carcasses. Endangered Species Research, 43, 395–408. 10.3354/esr01075

[ece38331-bib-0006] Beehner, J. C. , & Bergman, T. J. (2017). The next step for stress research in primates: To identify relationships between glucocorticoid secretion and fitness. Hormones and Behavior, 91, 68–83. 10.1016/j.yhbeh.2017.03.003 28284709

[ece38331-bib-0007] Behringer, V. , Deimel, C. , Hohmann, G. , Negrey, J. , Schaebs, F. S. , & Deschner, T. (2018). Applications for non‐invasive thyroid hormone measurements in mammalian ecology, growth, and maintenance. Hormones and Behavior, 105, 66–85. 10.1016/j.yhbeh.2018.07.011 30063897

[ece38331-bib-0008] Beldomenico, P. M. , & Begon, M. (2010). Disease spread, susceptibility and infection intensity: Vicious circles? Trends in Ecology & Evolution, 25, 21–27. 19782425 10.1016/j.tree.2009.06.015

[ece38331-bib-0009] Bellis, J. , Bourke, D. , Maschinski, J. , Heineman, K. , & Dalrymple, S. (2020). Climate suitability as a predictor of conservation translocation failure. Conservation Biology, 34, 1473–1481. 10.1111/cobi.13518 32304113

[ece38331-bib-0010] Bettridge, C. , Lehmann, J. , & Dunbar, R. (2010). Trade‐offs between time, predation risk and life history, and their implications for biogeography: A systems modelling approach with a primate case study. Ecological Modelling, 221, 777–790. 10.1016/j.ecolmodel.2009.11.017

[ece38331-bib-0011] Blas, J. , Bortolotti, G. R. , Tella, J. L. , Baos, R. , & Marchant, T. A. (2007). Stress response during development predicts fitness in a wild, long lived vertebrate. Proceedings of the National Academy of Sciences of the United States of America, 104, 8880–8884. 10.1073/pnas.0700232104 17517658 PMC1868653

[ece38331-bib-0012] Blus, L. J. (2011). DDT, DDD, and DDE in birds. In W. N. Beyer , G. H. Heinz , & A. W. Redmon‐Norwood (Eds.), Environmental contaminants in biota (pp. 425–446). CRC Press.

[ece38331-bib-0013] Bocherens, H. , Hofman‐Kamińska, E. , Drucker, D. G. , Schmölcke, U. , & Kowalczyk, R. (2015). European bison as a refugee species? Evidence from isotopic data on Early Holocene bison and other large herbivores in northern Europe. PLoS One, 10, e0115090. 10.1371/journal.pone.0115090 25671634 PMC4324907

[ece38331-bib-0014] Bonier, F. , Moore, I. T. , Martin, P. R. , & Robertson, R. J. (2009). The relationship between fitness and baseline glucocorticoids in a passerine bird. General and Comparative Endocrinology, 163, 208–213. 10.1016/j.ygcen.2008.12.013 19135999

[ece38331-bib-0015] Borbón‐García, A. , Reyes, A. , Vives‐Flórez, M. , & Caballero, S. (2017). Captivity shapes the gut microbiota of Andean bears: Insights into health surveillance. Frontiers in Microbiology, 8, 1316. 10.3389/fmicb.2017.01316 28751883 PMC5507997

[ece38331-bib-0016] Breuner, C. W. , Patterson, S. H. , & Hahn, T. P. (2008). In search of relationships between the acute adrenocortical response and fitness. General and Comparative Endocrinology, 157, 288–295. 10.1016/j.ygcen.2008.05.017 18602555

[ece38331-bib-0017] Brinkmann, L. , Gerken, M. , Hambly, C. , Speakman, J. R. , & Riek, A. (2016). Thyroid hormones correlate with field metabolic rate in ponies, Equus ferus caballus. Journal of Experimental Biology, 219, 2559–2566. 10.1242/jeb.138784 27312472

[ece38331-bib-0162] Britnell, J. A. , Lewis, R. N. , Elsner‐Gearing, F. , Harvey, N. , Stanbrook, E. , & Shultz, S. (2021). Species stereotypes as a result of unconscious research biases compromise conservation efficacy. Biological Conservation, 261, 109275. 10.1016/j.biocon.2021.109275

[ece38331-bib-0018] Brook, B. W. , O'Grady, J. J. , Chapman, A. P. , Burgman, M. A. , Akcakaya, H. R. , & Frankham, R. (2000). Predictive accuracy of population viability analysis in conservation biology. Nature, 404, 385–387. 10.1038/35006050 10746724

[ece38331-bib-0019] Brook, B. W. , Sodhi, N. S. , & Bradshaw, C. J. (2008). Synergies among extinction drivers under global change. Trends in Ecology & Evolution, 23, 453–460. 10.1016/j.tree.2008.03.011 18582986

[ece38331-bib-0020] Buckley, L. B. , Urban, M. C. , Angilletta, M. J. , Crozier, L. G. , Rissler, L. J. , & Sears, M. W. (2010). Can mechanism inform species’ distribution models? Ecology Letters, 13, 1041–1054. 10.1111/j.1461-0248.2010.01479.x 20482574

[ece38331-bib-0021] Burger, J. , & Gochfeld, M. (2004). Marine birds as sentinels of environmental pollution. EcoHealth, 1, 263–274. 10.1007/s10393-004-0096-4

[ece38331-bib-0163] Burger, J. R. , Hou, C. , & Brown, J. H. (2019). Toward a metabolic theory of life history. Proceedings of the National Academy of Sciences, 116(52), 26653–26661. 10.1073/pnas.1907702116 PMC6936346 31822607

[ece38331-bib-0022] Celi, P. , Verlhac, V. , Calvo, E. P. , Schmeisser, J. , & Kluenter, A.‐M. (2019). Biomarkers of gastrointestinal functionality in animal nutrition and health. Animal Feed Science and Technology, 250, 9–31. 10.1016/j.anifeedsci.2018.07.012

[ece38331-bib-0023] Champagne, C. D. , Kellar, N. M. , Trego, M. L. , Delehanty, B. , Boonstra, R. , Wasser, S. K. , Booth, R. K. , Crocker, D. E. , & Houser, D. S. (2018). Comprehensive endocrine response to acute stress in the bottlenose dolphin from serum, blubber, and feces. General and Comparative Endocrinology, 266, 178–193. 10.1016/j.ygcen.2018.05.015 29852162

[ece38331-bib-0024] Channell, R. , & Lomolino, M. V. (2000). Trajectories to extinction: Spatial dynamics of the contraction of geographical ranges. Journal of Biogeography, 27, 169–179. 10.1046/j.1365-2699.2000.00382.x

[ece38331-bib-0025] Chown, S. L. , & Gaston, K. J. (2008). Macrophysiology for a changing world. Proceedings of the Royal Society B: Biological Sciences, 275, 1469–1478. 10.1098/rspb.2008.0137 PMC2394563 18397867

[ece38331-bib-0026] Christiansen, F. , Rasmussen, M. H. , & Lusseau, D. (2013). Inferring activity budgets in wild animals to estimate the consequences of disturbances. Behavioral Ecology, 24, 1415–1425. 10.1093/beheco/art086

[ece38331-bib-0027] Clinchy, M. , Sheriff, M. J. , & Zanette, L. Y. (2013). Predator‐induced stress and the ecology of fear. Functional Ecology, 27, 56–65. 10.1111/1365-2435.12007

[ece38331-bib-0028] Clinchy, M. , Zanette, L. , Charlier, T. D. , Newman, A. E. , Schmidt, K. L. , Boonstra, R. , & Soma, K. K. (2011). Multiple measures elucidate glucocorticoid responses to environmental variation in predation threat. Oecologia, 166, 607–614. 10.1007/s00442-011-1915-2 21279653

[ece38331-bib-0029] Cooke, S. J. , Sack, L. , Franklin, C. E. , Farrell, A. P. , Beardall, J. , Wikelski, M. , & Chown, S. L. (2013). What is conservation physiology? Perspectives on an increasingly integrated and essential science. Conservation Physiology, 1, cot001. 10.1093/conphys/cot001 27293585 PMC4732437

[ece38331-bib-0030] Cooke, S. J. , & Suski, C. D. (2008). Ecological restoration and physiology: An overdue integration. BioScience, 58, 957–968. 10.1641/B581009

[ece38331-bib-0031] Costa‐e‐Sousa, R. H. , & Hollenberg, A. N. (2012). Minireview: The neural regulation of the hypothalamic‐pituitary‐thyroid axis. Endocrinology, 153, 4128–4135. 10.1210/en.2012-1467 22759379 PMC3423621

[ece38331-bib-0032] Costantini, D. , Marasco, V. , & Møller, A. P. (2011). A meta‐analysis of glucocorticoids as modulators of oxidative stress in vertebrates. Journal of Comparative Physiology B, 181, 447–456. 10.1007/s00360-011-0566-2 21416253

[ece38331-bib-0033] Cristóbal‐Azkarate, J. , Maréchal, L. , Semple, S. , Majolo, B. , & MacLarnon, A. (2016). Metabolic strategies in wild male Barbary macaques: Evidence from faecal measurement of thyroid hormone. Biology Letters, 12, 20160168. 10.1098/rsbl.2016.0168 27095269 PMC4881361

[ece38331-bib-0034] Davis, A. , Maney, D. , & Maerz, J. (2008). The use of leukocyte profiles to measure stress in vertebrates: A review for ecologists. Functional Ecology, 22, 760–772. 10.1111/j.1365-2435.2008.01467.x

[ece38331-bib-0035] Deelen, J. , Kettunen, J. , Fischer, K. , van der Spek, A. , Trompet, S. , Kastenmüller, G. , Boyd, A. , Zierer, J. , van den Akker, E. B. , Ala‐Korpela, M. , Amin, N. , Demirkan, A. , Ghanbari, M. , van Heemst, D. , Ikram, M. A. , van Klinken, J. B. , Mooijaart, S. P. , Peters, A. , Salomaa, V. , … Slagboom, P. E. (2019). A metabolic profile of all‐cause mortality risk identified in an observational study of 44,168 individuals. Nature Communications, 10, 1–8. 10.1038/s41467-019-11311-9 PMC6702196 31431621

[ece38331-bib-0036] DeRango, E. J. , Greig, D. J. , Gálvez, C. , Norris, T. A. , Barbosa, L. , Elorriaga‐Verplancken, F. R. , & Crocker, D. E. (2019). Response to capture stress involves multiple corticosteroids and is associated with serum thyroid hormone concentrations in Guadalupe fur seals (Arctocephalus philippii townsendi). Marine Mammal Science, 35, 72–92.

[ece38331-bib-0037] Dethlefsen, L. , McFall‐Ngai, M. , & Relman, D. A. (2007). An ecological and evolutionary perspective on human–microbe mutualism and disease. Nature, 449, 811–818. 10.1038/nature06245 17943117 PMC9464033

[ece38331-bib-0038] Dias, M. P. , Martin, R. , Pearmain, E. J. , Burfield, I. J. , Small, C. , Phillips, R. A. , Yates, O. , Lascelles, B. , Borboroglu, P. G. , & Croxall, J. P. (2019). Threats to seabirds: A global assessment. Biological Conservation, 237, 525–537. 10.1016/j.biocon.2019.06.033

[ece38331-bib-0039] Dickens, M. J. , Delehanty, D. J. , & Romero, L. M. (2010). Stress: An inevitable component of animal translocation. Biological Conservation, 143, 1329–1341. 10.1016/j.biocon.2010.02.032

[ece38331-bib-0040] Dickens, M. J. , & Romero, L. M. (2013). A consensus endocrine profile for chronically stressed wild animals does not exist. General and Comparative Endocrinology, 191, 177–189. 10.1016/j.ygcen.2013.06.014 23816765

[ece38331-bib-0041] Dunbar, R. I. , Korstjens, A. H. , & Lehmann, J. (2009). Time as an ecological constraint. Biological Reviews, 84, 413–429. 10.1111/j.1469-185X.2009.00080.x 19485986

[ece38331-bib-0042] Dunlap, K. D. (1995). External and internal influences on indices of physiological stress: II. Seasonal and size‐related variations in blood composition in free‐living lizards, Sceloporus occidentalis. Journal of Experimental Zoology, 272, 85–94. 10.1002/jez.1402720202 7622998

[ece38331-bib-0043] Dunlap, K. D. , & Wingfield, J. (1995). External and internal influences on indices of physiological stress. I. Seasonal and population variation in adrenocortical secretion of free‐living lizards, *Sceloporus occidentalis* . Journal of Experimental Zoology, 271, 36–46. 10.1002/jez.1402710105 7852947

[ece38331-bib-0044] Edwards, K. L. , Shultz, S. , Pilgrim, M. , & Walker, S. L. (2015). Irregular ovarian activity, body condition and behavioural differences are associated with reproductive success in female eastern black rhinoceros (Diceros bicornis michaeli). General and Comparative Endocrinology, 214, 186–194. 10.1016/j.ygcen.2014.07.026 25150145

[ece38331-bib-0045] Edwards, K. L. , Walker, S. L. , Bodenham, R. F. , Ritchie, H. , & Shultz, S. (2013). Associations between social behaviour and adrenal activity in female Barbary macaques: Consequences of study design. General and Comparative Endocrinology, 186, 72–79. 10.1016/j.ygcen.2013.02.023 23474330

[ece38331-bib-0046] Ellis, R. D. , McWhorter, T. J. , & Maron, M. (2012). Integrating landscape ecology and conservation physiology. Landscape Ecology, 27, 1–12. 10.1007/s10980-011-9671-6

[ece38331-bib-0047] Escribano‐Avila, G. , Pettorelli, N. , Virgós, E. , Lara‐Romero, C. , Lozano, J. , Barja, I. , Cuadra, F. S. , & Puerta, M. (2013). Testing Cort‐Fitness and Cort‐Adaptation hypotheses in a habitat suitability gradient for roe deer. Acta Oecologica, 53, 38–48. 10.1016/j.actao.2013.08.003

[ece38331-bib-0048] Ezenwa, V. O. , Jolles, A. E. , & O’Brien, M. P. (2009). A reliable body condition scoring technique for estimating condition in African buffalo. African Journal of Ecology, 47, 476–481. 10.1111/j.1365-2028.2008.00960.x

[ece38331-bib-0049] Fair, J. , Whitaker, S. , & Pearson, B. (2007). Sources of variation in haematocrit in birds. Ibis, 149, 535–552. 10.1111/j.1474-919X.2007.00680.x

[ece38331-bib-0050] Faith, J. T. (2012). Palaeozoological insights into management options for a threatened mammal: Southern Africa’s Cape mountain zebra (*Equus zebra zebra*). Diversity and Distributions, 18, 438–447. 10.1111/j.1472-4642.2011.00841.x

[ece38331-bib-0051] Fajardo, I. , Babiloni, G. , & Miranda, Y. (2000). Rehabilitated and wild barn owls (*Tyto alba*): Dispersal, life expectancy and mortality in Spain. Biological Conservation, 94, 287–295. 10.1016/S0006-3207(00)00003-3

[ece38331-bib-0052] Finkel, T. , & Holbrook, N. J. (2000). Oxidants, oxidative stress and the biology of ageing. Nature, 408, 239. 11089981 10.1038/35041687

[ece38331-bib-0053] Foley, C. A. H. , Papageorge, S. , & Wasser, S. K. (2001). Noninvasive stress and reproductive measures of social and ecological pressures in free‐ranging African elephants. Conservation Biology, 15, 1134–1142. 10.1046/j.1523-1739.2001.0150041134.x

[ece38331-bib-0054] Fretwell, S. D. (1969). On territorial behavior and other factors influencing habitat distribution in birds. Acta Biotheoretica, 19, 45–52. 10.1007/BF01601955

[ece38331-bib-0055] Fretwell, S. (1972). Populations in a seasonal environment. Monographs in Population Biology, 5, 1–217. 4680650

[ece38331-bib-0056] Gaillard, J. M. , Festa‐Bianchet, M. , Yoccoz, N. G. , Loison, A. , & Toigo, C. (2000). Temporal variation in fitness components and population dynamics of large herbivores. Annual Review of Ecology and Systematics, 31, 367–393. 10.1146/annurev.ecolsys.31.1.367

[ece38331-bib-0057] Gallagher, A. J. , Creel, S. , Wilson, R. P. , & Cooke, S. J. (2017). Energy landscapes and the landscape of fear. Trends in Ecology & Evolution, 32, 88–96. 10.1016/j.tree.2016.10.010 27814919

[ece38331-bib-0058] Ganswindt, A. , Münscher, S. , Henley, M. , Palme, R. , Thompson, P. , & Bertschinger, H. (2010). Concentrations of faecal glucocorticoid metabolites in physically injured free‐ranging African elephants *Loxodonta africana* . Wildlife Biology, 16, 323–332. 10.2981/09-081

[ece38331-bib-0059] Gaston, K. J. , Chown, S. L. , Calosi, P. , Bernardo, J. , Bilton, D. T. , Clarke, A. , Clusella‐Trullas, S. , Ghalambor, C. K. , Konarzewski, M. , Peck, L. S. , Porter, W. P. , Pörtner, H. O. , Rezende, E. L. , Schulte, P. M. , Spicer, J. I. , Stillman, J. H. , Terblanche, J. S. , & van Kleunen, M. (2009). Macrophysiology: A conceptual reunification. The American Naturalist, 174, 595–612. 10.1086/605982 19788354

[ece38331-bib-0160] Gersick, A. S. , & Rubenstein, D. I. (2017). Physiology modulates social flexibility and collective behaviour in equids and other large ungulates. Philosophical Transactions of the Royal Society B: Biological Sciences, 372(1727), 20160241. 10.1098/rstb.2016.0241 PMC5498301 28673917

[ece38331-bib-0060] Gilbert, J. A. , Blaser, M. J. , Caporaso, J. G. , Jansson, J. K. , Lynch, S. V. , & Knight, R. (2018). Current understanding of the human microbiome. Nature Medicine, 24, 392. 10.1038/nm.4517 PMC7043356 29634682

[ece38331-bib-0061] Gilbert, J. A. , Quinn, R. A. , Debelius, J. , Xu, Z. Z. , Morton, J. , Garg, N. , Jansson, J. K. , Dorrestein, P. C. , & Knight, R. (2016). Microbiome‐wide association studies link dynamic microbial consortia to disease. Nature, 535, 94–103. 10.1038/nature18850 27383984

[ece38331-bib-0062] Gobush, K. , Booth, R. , & Wasser, S. (2014). Validation and application of noninvasive glucocorticoid and thyroid hormone measures in free‐ranging Hawaiian monk seals. General and Comparative Endocrinology, 195, 174–182. 10.1016/j.ygcen.2013.10.020 24239792

[ece38331-bib-0063] Green, R. E. , Newton, I. , Shultz, S. , Cunningham, A. A. , Gilbert, M. , Pain, D. J. , & Prakash, V. (2004). Diclofenac poisoning as a cause of vulture population declines across the Indian subcontinent. Journal of Applied Ecology, 41, 793–800. 10.1111/j.0021-8901.2004.00954.x

[ece38331-bib-0064] Grueter, C. C. , Li, D. , Ren, B. , Wei, F. , Xiang, Z. , & van Schaik, C. P. (2009). Fallback foods of temperate‐living primates: A case study on snub‐nosed monkeys. American Journal of Physical Anthropology, 140, 700–715. 10.1002/ajpa.21024 19890849

[ece38331-bib-0065] Guo, Q. , Taper, M. , Schoenberger, M. , & Brandle, J. (2005). Spatial‐temporal population dynamics across species range: From centre to margin. Oikos, 108, 47–57. 10.1111/j.0030-1299.2005.13149.x

[ece38331-bib-0066] Harlow, H. J. , & Seal, U. S. (1981). Changes in hematology and metabolites in the serum and urine of the badger, *Taxidea taxus*, during food deprivation. Canadian Journal of Zoology, 59, 2123–2128.

[ece38331-bib-0067] Harting, A. L. , Barbieri, M. M. , Baker, J. D. , Mercer, T. A. , Johanos, T. C. , Robinson, S. J. , Littnan, C. L. , Colegrove, K. M. , & Rotstein, D. S. (2021). Population‐level impacts of natural and anthropogenic causes‐of‐death for Hawaiian monk seals in the main Hawaiian Islands. Marine Mammal Science, 37, 235–250. 10.1111/mms.12742

[ece38331-bib-0068] Hillegass, M. A. , Waterman, J. M. , & Roth, J. D. (2010). Parasite removal increases reproductive success in a social African ground squirrel. Behavioral Ecology, 21, 696–700. 10.1093/beheco/arq041

[ece38331-bib-0069] Hing, S. , Narayan, E. J. , Thompson, R. C. A. , & Godfrey, S. S. (2016). The relationship between physiological stress and wildlife disease: Consequences for health and conservation. Wildlife Research, 43, 51–60. 10.1071/WR15183

[ece38331-bib-0070] Hobbs, R. J. , Higgs, E. , Hall, C. M. , Bridgewater, P. , Chapin, F. S. , Ellis, E. C. , Ewel, J. J. , Hallett, L. M. , Harris, J. , Hulvey, K. B. , Jackson, S. T. , Kennedy, P. L. , Kueffer, C. , Lach, L. , Lantz, T. C. , Lugo, A. E. , Mascaro, J. , Murphy, S. D. , Nelson, C. R. , … Yung, L. (2014). Managing the whole landscape: Historical, hybrid, and novel ecosystems. Frontiers in Ecology and the Environment, 12, 557–564. 10.1890/130300

[ece38331-bib-0071] Hoffmann, M. , Hilton‐Taylor, C. , Angulo, A. , Böhm, M. , Brooks, T. M. , Butchart, S. H. M. , Carpenter, K. E. , Chanson, J. , Collen, B. , Cox, N. A. , Darwall, W. R. T. , Dulvy, N. K. , Harrison, L. R. , Katariya, V. , Pollock, C. M. , Quader, S. , Richman, N. I. , Rodrigues, A. S. L. , Tognelli, M. F. , … Stuart, S. N. (2010). The impact of conservation on the status of the world's vertebrates. Science, 330, 1503–1509. 10.1126/science.1194442 20978281

[ece38331-bib-0072] Holt, R. D. (2003). On the evolutionary ecology of species’ ranges. Evolutionary Ecology Research, 5, 159–178.

[ece38331-bib-0073] Holt, R. D. (2009). Bringing the Hutchinsonian niche into the 21st century: Ecological and evolutionary perspectives. Proceedings of the National Academy of Sciences of the United States of America, 106, 19659–19665. 10.1073/pnas.0905137106 19903876 PMC2780934

[ece38331-bib-0074] Hudson, P. J. (1986). The effect of a parasitic nematode on the breeding production of red grouse. The Journal of Animal Ecology, 55(1), 85–92. 10.2307/4694

[ece38331-bib-0075] Hudson, P. J. , Dobson, A. P. , & Newborn, D. (1998). Prevention of population cycles by parasite removal. Science, 282, 2256–2258. 10.1126/science.282.5397.2256 9856948

[ece38331-bib-0076] Ingala, M. R. , Becker, D. J. , Bak Holm, J. , Kristiansen, K. , & Simmons, N. B. (2019). Habitat fragmentation is associated with dietary shifts and microbiota variability in common vampire bats. Ecology and Evolution, 9, 6508–6523. 10.1002/ece3.5228 31236240 PMC6580296

[ece38331-bib-0077] Jachowski, D. , Slotow, R. , & Millspaugh, J. (2013). Delayed physiological acclimatization by African elephants following reintroduction. Animal Conservation, 16, 575–583. 10.1111/acv.12031

[ece38331-bib-0078] Jensen, A. A. , & Leffers, H. (2008). Emerging endocrine disrupters: Perfluoroalkylated substances. International Journal of Andrology, 31, 161–169. 10.1111/j.1365-2605.2008.00870.x 18315716

[ece38331-bib-0079] Johnson, J. B. , & Omland, K. S. (2004). Model selection in ecology and evolution. Trends in Ecology & Evolution, 19, 101–108. 10.1016/j.tree.2003.10.013 16701236

[ece38331-bib-0080] Kawecki, T. J. (2008). Adaptation to marginal habitats. Annual Review of Ecology, Evolution, and Systematics, 39, 321–342. 10.1146/annurev.ecolsys.38.091206.095622

[ece38331-bib-0081] Kearney, M. , & Porter, W. (2009). Mechanistic niche modelling: Combining physiological and spatial data to predict species’ ranges. Ecology Letters, 12, 334–350. 10.1111/j.1461-0248.2008.01277.x 19292794

[ece38331-bib-0082] Kerley, G. , Kowalczyk, R. , & Cromsigt, J. (2012). Conservation implications of the refugee species concept and the European bison: King of the forest or refugee in a marginal habitat? Ecography, 35, 519–529. 10.1111/j.1600-0587.2011.07146.x

[ece38331-bib-0083] Kitaysky, A. S. , Kitaiskaia, E. V. , Piatt, J. F. , & Wingfield, J. C. (2006). A mechanistic link between chick diet and decline in seabirds? Proceedings of the Royal Society B: Biological Sciences, 273, 445–450. 10.1098/rspb.2005.3351 PMC1560207 16615211

[ece38331-bib-0084] Koprivnikar, J. , & Penalva, L. (2015). Lesser of two evils? Foraging choices in response to threats of predation and parasitism. PLoS One, 10, e0116569. 10.1371/journal.pone.0116569 25635765 PMC4312073

[ece38331-bib-0085] Korstjens, A. H. , & Dunbar, R. I. M. (2007). Time constraints limit group sizes and distribution in red and black‐and‐white Colobus. International Journal of Primatology, 28, 551–575. 10.1007/s10764-007-9148-2 PMC6182722 30369685

[ece38331-bib-0086] Korstjens, A. H. , Lehmann, J. , & Dunbar, R. (2010). Resting time as an ecological constraint on primate biogeography. Animal Behaviour, 79, 361–374. 10.1016/j.anbehav.2009.11.012

[ece38331-bib-0087] Landman, M. , Schoeman, D. S. , & Kerley, G. I. H. (2013). Shift in black rhinoceros diet in the presence of elephant: Evidence for competition? PLoS One, 8, e69771. 10.1371/journal.pone.0069771 23874997 PMC3714249

[ece38331-bib-0088] Laundré, J. W. , Hernández, L. , & Altendorf, K. B. (2001). Wolves, elk, and bison: Reestablishing the “landscape of fear” in Yellowstone National Park, USA. Canadian Journal of Zoology, 79, 1401–1409. 10.1139/z01-094

[ece38331-bib-0089] Lea, J. M. D. , Walker, S. L. , Kerley, G. I. H. , Jackson, J. , Matevich, S. C. , & Shultz, S. (2018). Non‐invasive physiological markers demonstrate link between habitat quality, adult sex ratio and poor population growth rate in a vulnerable species, the Cape mountain zebra. Functional Ecology, 32, 300–312. 10.1111/1365-2435.13000

[ece38331-bib-0090] Madliger, C. L. , Love, O. P. , Hultine, K. R. , & Cooke, S. J. (2018). The conservation physiology toolbox: Status and opportunities. Conservation Physiology, 6, coy029. 10.1093/conphys/coy029 29942517 PMC6007632

[ece38331-bib-0091] Manire, C. A. , Walsh, C. J. , Rhinehart, H. L. , Colbert, D. E. , Noyes, D. R. , & Luer, C. A. (2003). Alterations in blood and urine parameters in two Florida manatees (*Trichechus manatus latirostris*) from simulated conditions of release following rehabilitation. Zoo Biology, 22, 103–120. 10.1002/zoo.10074

[ece38331-bib-0092] Mao, R. , Xiao, Y.‐L. , Gao, X. , Chen, B.‐L. , He, Y. , Yang, L. , Hu, P.‐J. , & Chen, M.‐H. (2012). Fecal calprotectin in predicting relapse of inflammatory bowel diseases: A meta‐analysis of prospective studies. Inflammatory Bowel Diseases, 18, 1894–1899. 10.1002/ibd.22861 22238138

[ece38331-bib-0093] McEwen, B. S. , & Wingfield, J. C. (2003). The concept of allostasis in biology and biomedicine. Hormones and Behavior, 43, 2–15. 10.1016/S0018-506X(02)00024-7 12614627

[ece38331-bib-0094] McGill, B. J. , Enquist, B. J. , Weiher, E. , & Westoby, M. (2006). Rebuilding community ecology from functional traits. Trends in Ecology & Evolution, 21, 178–185. 10.1016/j.tree.2006.02.002 16701083

[ece38331-bib-0095] McKay, D. M. (2009). The therapeutic helminth? Trends in Parasitology, 25, 109–114. 10.1016/j.pt.2008.11.008 19167926

[ece38331-bib-0096] McLennan, M. R. , Howell, C. P. , Bardi, M. , & Heistermann, M. (2019). Are human‐dominated landscapes stressful for wild chimpanzees (*Pan troglodytes*)? Biological Conservation, 233, 73–82. 10.1016/j.biocon.2019.02.028

[ece38331-bib-0097] Millspaugh, J. J. , & Washburn, B. E. (2004). Use of fecal glucocorticoid metabolite measures in conservation biology research: Considerations for application and interpretation. General and Comparative Endocrinology, 138, 189–199. 10.1016/j.ygcen.2004.07.002 15364201

[ece38331-bib-0098] Milot, E. , Cohen, A. A. , Vézina, F. , Buehler, D. M. , Matson, K. D. , & Piersma, T. (2014). A novel integrative method for measuring body condition in ecological studies based on physiological dysregulation. Methods in Ecology and Evolution, 5, 146–155. 10.1111/2041-210X.12145

[ece38331-bib-0099] Moberg, G. P. (2000). Biological response to stress: Implications for animal welfare. In G. P. Moberg & J. A. Mench (Eds.), The biology of animal stress: Basic principles and implications for animal welfare (vol. 1, pp. 21). CABI Publishing.

[ece38331-bib-0100] Mueller, M. , Sternecker, K. , Milz, S. , & Geist, J. (2020). Assessing turbine passage effects on internal fish injury and delayed mortality using X‐ray imaging. PeerJ, 8, e9977. 10.7717/peerj.9977 32995098 PMC7501806

[ece38331-bib-0101] Noguera, J. C. , Aira, M. , Pérez‐Losada, M. , Domínguez, J. , & Velando, A. (2018). Glucocorticoids modulate gastrointestinal microbiome in a wild bird. Royal Society Open Science, 5, 171743. 10.1098/rsos.171743 29765642 PMC5936907

[ece38331-bib-0102] Nuñez, C. M. V. , Adelman, J. S. , Smith, J. , Gesquiere, L. R. , & Rubenstein, D. I. (2014). Linking social environment and stress physiology in feral mares (*Equus caballus*): Group transfers elevate fecal cortisol levels. General and Comparative Endocrinology, 196, 26–33. 10.1016/j.ygcen.2013.11.012 24275609

[ece38331-bib-0103] O’Dwyer, K. , Dargent, F. , Forbes, M. R. , & Koprivnikar, J. (2020). Parasite infection leads to widespread glucocorticoid hormone increases in vertebrate hosts: A meta‐analysis. Journal of Animal Ecology, 89, 519–529. 10.1111/1365-2656.13123 31622499

[ece38331-bib-0104] Osorio‐Olvera, L. , Soberón, J. , & Falconi, M. (2019). On population abundance and niche structure. Ecography, 42, 1415–1425. 10.1111/ecog.04442

[ece38331-bib-0105] Ouyang, J. Q. , de Jong, M. , Hau, M. , Visser, M. E. , van Grunsven, R. H. , & Spoelstra, K. (2015). Stressful colours: Corticosterone concentrations in a free‐living songbird vary with the spectral composition of experimental illumination. Biology Letters, 11, 20150517. 10.1098/rsbl.2015.0517 26311159 PMC4571683

[ece38331-bib-0106] Pallin, L. J. , Baker, C. S. , Steel, D. , Kellar, N. M. , Robbins, J. , Johnston, D. W. , Nowacek, D. P. , Read, A. J. , & Friedlaender, A. S. (2018). High pregnancy rates in humpback whales (Megaptera novaeangliae) around the Western Antarctic Peninsula, evidence of a rapidly growing population. Royal Society Open Science, 5, 180017. 29892441 10.1098/rsos.180017 PMC5990787

[ece38331-bib-0107] Parsons, N. J. , Vanstreels, R. E. , & Schaefer, A. M. (2018). Prognostic indicators of rehabilitation outcomes for adult African penguins (*Spheniscus demersus*). Journal of Wildlife Diseases, 54, 54–65. 10.7589/2017-06-146 29059011

[ece38331-bib-0108] Pearson, R. G. , & Dawson, T. P. (2003). Predicting the impacts of climate change on the distribution of species: Are bioclimate envelope models useful? Global Ecology and Biogeography, 12, 361–371. 10.1046/j.1466-822X.2003.00042.x

[ece38331-bib-0109] Pedersen, A. B. , & Fenton, A. (2007). Emphasizing the ecology in parasite community ecology. Trends in Ecology & Evolution, 22, 133–139. 10.1016/j.tree.2006.11.005 17137676

[ece38331-bib-0110] Pironon, S. , Papuga, G. , Villellas, J. , Angert, A. L. , García, M. B. , & Thompson, J. D. (2017). Geographic variation in genetic and demographic performance: New insights from an old biogeographical paradigm. Biological Reviews, 92, 1877–1909. 10.1111/brv.12313 27891813

[ece38331-bib-0111] Pottinger, T. G. (2003). Interactions of endocrine‐disrupting chemicals with stress responses in wildlife. Pure and Applied Chemistry, 75, 2321–2333. 10.1351/pac200375112321

[ece38331-bib-0112] Pulliam, H. R. , & Danielson, B. J. (1991). Sources, sinks, and habitat selection: A landscape perspective on population dynamics. The American Naturalist, 137, S50–S66.

[ece38331-bib-0113] Rattner, B. A. , Eroschenko, V. P. , Fox, G. A. , Fry, D. M. , & Gorsline, J. (1984). Avian endocrine responses to environmental pollutants. Journal of Experimental Zoology, 232, 683–689. 10.1002/jez.1402320337 6394705

[ece38331-bib-0114] Reneerkens, J. , Piersma, T. , & Ramenofsky, M. (2002). An experimental test of the relationship between temporal variability of feeding opportunities and baseline levels of corticosterone in a shorebird. Journal of Experimental Zoology, 293, 81–88. 10.1002/jez.10113 12115922

[ece38331-bib-0115] Reuter, H. O. , & Adcock, K. (1998). Standardised body condition scoring system for black rhinoceros (*Diceros bicornis*). Pachyderm, 26, 116–121.

[ece38331-bib-0116] Rolland, R. M. , McLellan, W. A. , Moore, M. J. , Harms, C. A. , Burgess, E. A. , & Hunt, K. E. (2017). Fecal glucocorticoids and anthropogenic injury and mortality in North Atlantic right whales *Eubalaena glacialis* . Endangered Species Research, 34, 417–429. 10.3354/esr00866

[ece38331-bib-0117] Rowe, C. L. (2008). “The calamity of so long life”: Life histories, contaminants, and potential emerging threats to long‐lived vertebrates. BioScience, 58, 623–631. 10.1641/B580709

[ece38331-bib-0118] Sack, A. , Butler, E. , Cowen, P. , & Lewbart, G. A. (2017). Morbidity and mortality of wild turtles at a North Carolina wildlife clinic: A 10‐year retrospective. Journal of Zoo and Wildlife Medicine, 48, 716–724. 10.1638/2016-0053.1 28920820

[ece38331-bib-0119] Santini, L. , Pironon, S. , Maiorano, L. , & Thuiller, W. (2019). Addressing common pitfalls does not provide more support to geographical and ecological abundant‐centre hypotheses. Ecography, 42, 696–705. 10.1111/ecog.04027

[ece38331-bib-0120] Sapolsky, R. M. (1992). Cortisol concentrations and the social significance of rank instability among wild baboons. Psychoneuroendocrinology, 17, 701–709. 10.1016/0306-4530(92)90029-7 1287688

[ece38331-bib-0121] Scheele, B. C. , Foster, C. N. , Banks, S. C. , & Lindenmayer, D. B. (2017). Niche contractions in declining species: Mechanisms and consequences. Trends in Ecology & Evolution, 32, 346–355. 10.1016/j.tree.2017.02.013 28284374

[ece38331-bib-0122] Scheun, J. , Ludynia, K. , Snyman, A. , & Ganswindt, A. (2021). Non‐invasive hormone monitoring as a robust method for determining adrenocortical activity in injured, emaciated and oil‐contaminated African penguins undergoing rehabilitation. General and Comparative Endocrinology, 303, 113703. 33359663 10.1016/j.ygcen.2020.113703

[ece38331-bib-0123] Schlotz, W. , Hammerfald, K. , Ehlert, U. , & Gaab, J. (2011). Individual differences in the cortisol response to stress in young healthy men: Testing the roles of perceived stress reactivity and threat appraisal using multiphase latent growth curve modeling. Biological Psychology, 87, 257–264. 10.1016/j.biopsycho.2011.03.005 21419825

[ece38331-bib-0124] Schroeder, H. W. Jr. , & Cavacini, L. (2010). Structure and function of immunoglobulins. Journal of Allergy and Clinical Immunology, 125, S41–S52. 10.1016/j.jaci.2009.09.046 PMC3670108 20176268

[ece38331-bib-0125] Schwindt, A. R. , Winkelman, D. L. , Keteles, K. , Murphy, M. , & Vajda, A. M. (2014). An environmental oestrogen disrupts fish population dynamics through direct and transgenerational effects on survival and fecundity. Journal of Applied Ecology, 51, 582–591. 10.1111/1365-2664.12237

[ece38331-bib-0161] Seebacher, F. , & Krause, J. (2017). Physiological mechanisms underlying animal social behaviour. Philosophical Transactions of the Royal Society B: Biological Sciences, 372(1727), 20160231. 10.1098/rstb.2016.0231 PMC5498293 28673909

[ece38331-bib-0126] Semeniuk, C. A. , Bourgeon, S. , Smith, S. L. , & Rothley, K. D. (2009). Hematological differences between stingrays at tourist and non‐visited sites suggest physiological costs of wildlife tourism. Biological Conservation, 142, 1818–1829. 10.1016/j.biocon.2009.03.022

[ece38331-bib-0127] Shepard, E. L. C. , Wilson, R. P. , Rees, W. G. , Grundy, E. , Lambertucci, S. A. , & Vosper, S. B. (2013). Energy landscapes shape animal movement ecology. The American Naturalist, 182, 298–312. 10.1086/671257 23933722

[ece38331-bib-0128] Shultz, S. , Baral, H. S. , Charman, S. , Cunningham, A. A. , Das, D. , Ghalsasi, G. R. , Goudar, M. S. , Green, R. E. , Jones, A. , Nighot, P. , Pain, D. J. , & Prakash, V. (2004). Diclofenac poisoning is widespread in declining vulture populations across the Indian subcontinent. Proceedings of the Royal Society of London. Series B: Biological Sciences, 271, S458–S460. 10.1098/rsbl.2004.0223 15801603 PMC1810094

[ece38331-bib-0129] Sies, H. (1991). Oxidative stress: From basic research to clinical application. The American Journal of Medicine, 91, S31–S38. 10.1016/0002-9343(91)90281-2 1928209

[ece38331-bib-0130] Singer, A. C. , Shaw, H. , Rhodes, V. , & Hart, A. (2016). Review of antimicrobial resistance in the environment and its relevance to environmental regulators. Frontiers in Microbiology, 7, 1728. 10.3389/fmicb.2016.01728 27847505 PMC5088501

[ece38331-bib-0131] Slos, S. , & Stoks, R. (2008). Predation risk induces stress proteins and reduces antioxidant defense. Functional Ecology, 22, 637–642. 10.1111/j.1365-2435.2008.01424.x

[ece38331-bib-0132] Sommer, F. , & Bäckhed, F. (2013). The gut microbiota—masters of host development and physiology. Nature Reviews Microbiology, 11, 227–238. 10.1038/nrmicro2974 23435359

[ece38331-bib-0133] Sopinka, N. M. , Donaldson, M. R. , O’Connor, C. M. , Suski, C. D. , & Cooke, S. J. (2016). Stress indicators in fish. Fish physiology, 35, 405–462.

[ece38331-bib-0134] Sparks, A. M. , Watt, K. , Sinclair, R. , Pilkington, J. G. , Pemberton, J. M. , Johnston, S. E. , McNeilly, T. N. , & Nussey, D. H. (2018). Natural selection on antihelminth antibodies in a wild mammal population. The American Naturalist, 192, 745–760. 10.1086/700115 30444657

[ece38331-bib-0135] Støen, O.‐G. , Ordiz, A. , Evans, A. L. , Laske, T. G. , Kindberg, J. , Fröbert, O. , Swenson, J. E. , & Arnemo, J. M. (2015). Physiological evidence for a human‐induced landscape of fear in brown bears (*Ursus arctos*). Physiology & Behavior, 152, 244–248. 10.1016/j.physbeh.2015.09.030 26476156

[ece38331-bib-0136] Stumpf, R. M. , Gomez, A. , Amato, K. R. , Yeoman, C. J. , Polk, J. , Wilson, B. A. , Nelson, K. E. , White, B. , & Leigh, S. R. (2016). Microbiomes, metagenomics, and primate conservation: New strategies, tools, and applications. Biological Conservation, 199, 56–66. 10.1016/j.biocon.2016.03.035

[ece38331-bib-0137] Supali, T. , Verweij, J. J. , Wiria, A. E. , Djuardi, Y. , Hamid, F. , Kaisar, M. M. M. , Wammes, L. J. , Lieshout, L. V. , Luty, A. J. F. , Sartono, E. , & Yazdanbakhsh, M. (2010). Polyparasitism and its impact on the immune system. International Journal for Parasitology, 40, 1171–1176. 10.1016/j.ijpara.2010.05.003 20580905

[ece38331-bib-0138] Tarszisz, E. , Dickman, C. R. , & Munn, A. J. (2014). Physiology in conservation translocations. Conservation Physiology, 2, cou054. 10.1093/conphys/cou054 27293675 PMC4732500

[ece38331-bib-0139] Tartu, S. , Gabrielsen, G. W. , Blévin, P. , Ellis, H. , Bustnes, J. O. , Herzke, D. , & Chastel, O. (2014). Endocrine and fitness correlates of long‐chain perfluorinated carboxylates exposure in Arctic breeding black‐legged kittiwakes. Environmental Science & Technology, 48, 13504–13510. 10.1021/es503297n 25369114

[ece38331-bib-0140] Todgham, A. E. , & Stillman, J. H. (2013). Physiological responses to shifts in multiple environmental stressors: Relevance in a changing world. Integrative and Comparative Biology, 53, 539–544. 10.1093/icb/ict086 23892371

[ece38331-bib-0141] Trevelline, B. K. , Fontaine, S. S. , Hartup, B. K. , & Kohl, K. D. (2019). Conservation biology needs a microbial renaissance: A call for the consideration of host‐associated microbiota in wildlife management practices. Proceedings of the Royal Society B: Biological Sciences, 286, 20182448. 10.1098/rspb.2018.2448 PMC6364583 30963956

[ece38331-bib-0142] Tripp, K. M. , Verstegen, J. P. , Deutsch, C. J. , Bonde, R. K. , Wit, M. D. , Manire, C. A. , Gaspard, J. , & Harr, K. E. (2011). Evaluation of adrenocortical function in Florida manatees (*Trichechus manatus latirostris*). Zoo Biology, 30, 17–31. 20187090 10.1002/zoo.20311

[ece38331-bib-0143] van de Crommenacker, J. , Hammers, M. , van der Woude, J. , Louter, M. , Santema, P. , Richardson, D. S. , & Komdeur, J. (2017). Oxidative status and fitness components in the Seychelles warbler. Functional Ecology, 31, 1210–1219. 10.1111/1365-2435.12861

[ece38331-bib-0144] van de Crommenacker, J. , Komdeur, J. , Burke, T. , & Richardson, D. S. (2011). Spatio‐temporal variation in territory quality and oxidative status: A natural experiment in the Seychelles warbler (*Acrocephalus sechellensis*). Journal of Animal Ecology, 80, 668–680. 10.1111/j.1365-2656.2010.01792.x PMC3107423 21198588

[ece38331-bib-0145] van de Crommenacker, J. , Richardson, D. S. , Koltz, A. M. , Hutchings, K. , & Komdeur, J. (2012). Parasitic infection and oxidative status are associated and vary with breeding activity in the Seychelles warbler. Proceedings of the Royal Society B: Biological Sciences, 279, 1466–1476. 10.1098/rspb.2011.1865 PMC3282338 22048952

[ece38331-bib-0146] Van Meter, P. E. , French, J. A. , Dloniak, S. M. , Watts, H. E. , Kolowski, J. M. , & Holekamp, K. E. (2009). Fecal glucocorticoids reflect socio‐ecological and anthropogenic stressors in the lives of wild spotted hyenas. Hormones and Behavior, 55, 329–337. 10.1016/j.yhbeh.2008.11.001 19056392 PMC2987620

[ece38331-bib-0147] Van Rheenen, P. F. , Van de Vijver, E. , & Fidler, V. (2010). Faecal calprotectin for screening of patients with suspected inflammatory bowel disease: Diagnostic meta‐analysis. BMJ, 341, c3369. 10.1136/bmj.c3369 20634346 PMC2904879

[ece38331-bib-0148] Viijoen, J. J. , Ganswindt, A. , du Toit, J. T. , & Langbauer, W. R. (2008). Translocation stress and faecal glucocorticoid metabolite levels in free‐ranging African savanna elephants. South African Journal of Wildlife Research, 38, 146–152. 10.3957/0379-4369-38.2.146

[ece38331-bib-0149] Violle, C. , Navas, M. L. , Vile, D. , Kazakou, E. , Fortunel, C. , Hummel, I. , & Garnier, E. (2007). Let the concept of trait be functional! Oikos, 116, 882–892. 10.1111/j.0030-1299.2007.15559.x

[ece38331-bib-0150] Vynne, C. , Booth, R. K. , & Wasser, S. K. (2014). Physiological implications of landscape use by free‐ranging maned wolves (*Chrysocyon brachyurus*) in Brazil. Journal of Mammalogy, 95, 696–706.

[ece38331-bib-0151] Walshe, N. , Duggan, V. , Cabrera‐Rubio, R. , Crispie, F. , Cotter, P. , Feehan, O. , & Mulcahy, G. (2019). Removal of adult cyathostomins alters faecal microbiota and promotes an inflammatory phenotype in horses. International Journal for Parasitology, 49, 489–500. 10.1016/j.ijpara.2019.02.003 30986403

[ece38331-bib-0152] Wasser, S. K. , Lundin, J. I. , Ayres, K. , Seely, E. , Giles, D. , Balcomb, K. , Hempelmann, J. , Parsons, K. , & Booth, R. (2017). Population growth is limited by nutritional impacts on pregnancy success in endangered Southern Resident killer whales (*Orcinus orca*). PLoS One, 12, e0179824. 10.1371/journal.pone.0179824 28662095 PMC5491047

[ece38331-bib-0153] Watt, K. A. , Nussey, D. H. , Maclellan, R. , Pilkington, J. G. , & McNeilly, T. N. (2016). Fecal antibody levels as a noninvasive method for measuring immunity to gastrointestinal nematodes in ecological studies. Ecology and Evolution, 6, 56–67. 10.1002/ece3.1858 26811774 PMC4716500

[ece38331-bib-0154] Weinstein, S. B. , Buck, J. C. , & Young, H. S. (2018). A landscape of disgust. Science, 359, 1213–1214. 10.1126/science.aas8694 29590062

[ece38331-bib-0155] Wessling, E. G. , Deschner, T. , Mundry, R. , Pruetz, J. D. , Wittig, R. M. , & Kühl, H. S. (2018). Seasonal variation in physiology challenges the notion of chimpanzees (*Pan troglodytes* verus) as a forest‐adapted species. Frontiers in Ecology and Evolution, 6, 60. 10.3389/fevo.2018.00060

[ece38331-bib-0156] Wilson, A. E. , Wismer, D. , Stenhouse, G. , Coops, N. C. , & Janz, D. M. (2021). Landscape condition influences energetics, reproduction, and stress biomarkers in grizzly bears. Scientific Reports, 11, 1–16. 10.1038/s41598-021-91595-4 34108541 PMC8190091

[ece38331-bib-0157] Wolf, T. E. , Valades, G. B. , Simelane, P. , Bennett, N. C. , & Ganswindt, A. (2018). The relationship between physical injury, body condition and stress‐related hormone concentrations in free‐ranging giraffes. Wildlife Biology, 1, 1–6.

[ece38331-bib-0158] Wong, B. , & Candolin, U. (2015). Behavioral responses to changing environments. Behavioral Ecology, 26, 665–673. 10.1093/beheco/aru183

[ece38331-bib-0159] Zaneveld, J. R. , McMinds, R. , & Thurber, R. V. (2017). Stress and stability: Applying the Anna Karenina principle to animal microbiomes. Nature Microbiology, 2, 1–8. 10.1038/nmicrobiol.2017.121 28836573

